# Analgesic efficacy of the bilateral erector spinae plane block for colorectal surgery: a randomized controlled trial

**DOI:** 10.1186/s44158-022-00073-4

**Published:** 2022-10-21

**Authors:** Ozgenur Kekul, Yasemin Burcu Ustun, Cengiz Kaya, Esra Turunç, Burhan Dost, Sezgin Bilgin, Fatih Ozkan

**Affiliations:** Department of Anesthesiology and Reanimation, Faculty of Medicine, Ondokuz Mayis University, Samsun, Turkey

**Keywords:** Pain, Colorectal surgery, Erector spinae plane block, Regional anesthesia

## Abstract

**Background:**

Colorectal cancer is quite common, and surgery is the most effective treatment for most patients. However, postoperative pain management is generally inadequate in most patients. This study aimed to determine the effect of ultrasonography (USG)-guided preemptive erector spina plan block (ESPB), as part of multimodal analgesia, on postoperative analgesia in patients undergoing colorectal cancer surgery.

**Methods:**

This is a prospective, randomized, single-blind trial. This study included 60 patients (ASA I-II) who underwent colorectal surgery at the hospital of Ondokuz Mayis University. The patients were divided into the ESP group and control group. Intraoperatively, all patients were administered intravenous tenoxicam (20 mg) and paracetamol (1 g) as part of multimodal analgesia. Intravenous morphine via patient-controlled analgesia was administered in all groups postoperatively. The primary outcome was the total morphine consumption in the first 24 h after surgery. The secondary outcomes included visual analog scale pain scores at rest and coughing and deep inspiration in the first 24 h and at 3 months postoperatively; number of patients requesting rescue analgesia; incidence of nausea and vomiting and need for antiemetics; intraoperative remifentanil consumption; postoperative first oral intake; time to first urination, first defecation, and first mobilization; hospitalization time; and incidence of pruritus.

**Results:**

Morphine consumption in the first 6 h postoperatively, total amount of morphine consumed in the first 24 h postoperatively, pain scores, intraoperative remifentanil consumption, incidence of pruritus, and postoperative antiemetic requirement were lower in the ESP group than in the control group. First defecation time and hospitalization time were shorter in the block group.

**Conclusions:**

As a part of multimodal analgesia, ESPB reduced postoperative opioid consumption and pain scores in the early postoperative period and in the 3rd month.

## Introduction

Colorectal cancer is the third most common cancer globally. As a result of the increase in the incidence of colorectal cancer, colorectal cancer surgery and postoperative pain management have become important [1]. The Society for Enhanced Recovery after Surgery (ERAS®) recommends effective perioperative pain control to improve patient outcomes. The goal of pain management is to alleviate suffering, gain early mobilization after surgery, reduce hospital stay, and improve patient satisfaction and functional recovery [2]. Laparoscopic or laparotomic methods can be applied in colorectal surgery. While incision-related somatic pain and visceral pain are observed in laparotomy surgery, somatic pain, visceral pain, pneumoperitoneum-related pain, and shoulder pain may be observed in laparoscopic surgery due to trocar insertion [3]. The efficacy of neuraxial and paravertebral blocks in pain management following both surgical methods has been demonstrated. These methods are not only difficult to apply but also they may result in severe complications such as hypotension, bradycardia, motor blockade, urinary retention, and spinal hematoma [4]. Erector spinae plane block (ESPB) was first described for thoracic neuropathic pain relief in 2016 by Forero et al. [5]. It was observed that the local anesthetic agent reached the epidural and paravertebral areas through the costotransverse foramen with ESPB. It has been reported as an interfascial block that is easy to implement and has low complication rates in the literature, in addition to providing visceral and somatic analgesia [6].

This study aimed to evaluate the effects of ultrasound-guided ESPB, used as part of multimodal analgesia, on the postoperative 24-h morphine consumption and pain scores (up to 3 months) in patients undergoing colorectal surgery.

## Methods

The study was approved by the Ethics Committee of Ondokuz Mayıs University School of Medicine (approval no: 2020/508 19-AKD-158). It was registered on ClinicalTrials.gov (NCT05256953). It is a single-center, prospective, randomized, controlled, parallel-group, and single-blind study. Written informed consent was obtained from all participants for the interventions before including them. The participants were included in the study between January and September 2021.

### Study population

 Patients aged 18–65 years who underwent colorectal surgery, and who had American Society of Anesthesiologists (ASA) physical status classification I-II were included in the study. The exclusion criteria were as follows: history of colorectal surgery excluding diagnostic biopsies, coagulopathy, bleeding disorder, injection site infection, allergy to local anesthetics, pregnancy, or psychiatric disorders (depression, bipolar disorder, schizophreniform disorder, or history of antipsychotic drug use); history of opioid use (longer than 4 weeks); body mass index of > 35 kg/m^2^; block failure results of dermatomal examination performed after block application; and inability to be contacted via telephone for inquiry about pain scores within 3 months.

After the procedure, the sensory block (T6–L1 dermatomes) was checked by an anesthesiologist independent of the physician who applied it at the level of the bilateral mid-axillary line with a pinprick test (27 G hypodermic needle) applied every 5 min (0 = no sensory block; 1 = the presence of tactile sensation but no pain; 2 = no tactile sensation and pain). ESPB was defined as successful for patients with a sensory block score of 1 and above.

### Randomization and blinding

Patients were randomized to the ESPB and control groups, each consisting of 30 patients. Randomization was performed with computer-generated random numbers using Statistical Product and Service Solutions (SPSS version 23.0, IBM, New York, USA). Patient codes were placed in sequentially numbered, opaque, and sealed envelopes by a physician blinded to the study. An independent assistant who did not take part in the study opened a sealed envelope 1 h before the surgery and informed the anesthesiologist about the block method to be applied. These random numbers were also used during follow-up and analysis of patient data. While the investigators and outcome assessors were blinded to the intervention applied, due to the nature of the study, the anesthesiologist who administered the block and the patients were not blinded to the group distribution.

### Block procedure

ASA standard monitoring procedures (electrocardiograph and noninvasive arterial pressure and peripheral oxygen saturation measurements) were applied to the patients before the procedure; oxygen support was provided with a simple oxygen mask at a rate of 3 l/min. All patients were sedated by administering 0.02 mg/kg of midazolam. The imaginary line passing through the bilateral spina scapula level of the patients in the sitting position was defined as the T4 level, and it was located by palpation towards the caudal aspect. After taking the necessary sterilization measures, the transverse process of the T9 vertebrae and the erector spinae muscle group was visualized in the parasagittal plane using an ultrasound (USG) (2.5–5 MHz, MyLabFivePortable Ultrasound, UK) convex probe. Two milliliters of 2% lidocaine was injected into the input port of the block needle (21G 100-mm-short beveled needle, Stimuplex Ultra 360®, Braun, Germany). The plane between the erector spinae and transverse process was accessed with the block needle in the craniocaudal direction (in plane). Twenty milliliters of 0.25% bupivacaine was injected following hydrodissection. The procedure was repeated the same way on the other side.

### Anesthesia management

Monitoring by electrocardiogram, capnography was performed, and peripheral oxygen saturation, invasive arterial pressure, and bi-spectral index (Covidien, Minneapolis, MN, USA) were assessed in the operating room. After anesthesia induction with propofol (1.5–2.5 mg/kg) and remifentanil (1 µg/kg IV bolus for 30–60 s, followed by 0.25 µg/kg/min), tracheal intubation was performed with rocuronium (0.6 mg/kg). Intravenous infusion of Ringer’s lactate solution (5–7 ml/kg/h) was initiated in all patients. General anesthesia was maintained with sevoflurane and O2/air (fraction of inspired oxygen: 0.40). Caution was exercised to maintain the depth of anesthesia (*BIS* 40–60). The remifentanil infusion rate was changed according to the mean arterial pressure to maintain the heart rate within ± 20% of the preoperative values. At the end of the surgery, rocuronium’s effect was reversed with 2 mg/kg sugammadex. For postoperative nausea and vomiting (PONV) prophylaxis, the patients were administered 0.1 mg/kg IV dexamethasone before induction and 1.5 mg IV granisetron 20 min before the conclusion of the case. The patients with five-stage verbal descriptive nausea-vomiting scores of ≥ 2 (0 = no nausea or vomiting; 1 = mild nausea, no vomiting; 2 = moderate nausea, no vomiting; 3 = vomiting once; 4 = vomiting multiple times) during their follow-up in the recovery room were administered granisetron (1.5 mg), and the frequency of antiemetic use was recorded. Itching was monitored using the pruritus visual analog scale (VAS) (P-VAS) (0 = no pruritus, < 4 points = mild pruritus, ≥ 4 to < 7 points = moderate pruritus, ≥ 7 to < 9 points = severe pruritus, and ≥ 9 points = very severe pruritus). Patients with P-VAS of ≥ 4 were considered to have itching and were administered 50 mg IV diphenhydramine.

### Analgesia management

The VAS score (0 points = no pain, 10 points = worst pain imaginable) was explained to the patients during the preoperative period. In addition, information about the patient-controlled analgesia (PCA) device was provided. The patients were informed that they could request analgesics from the PCA device if their VAS was > 3 at rest in the recovery unit. All patients were administered tenoxicam (20 mg IV) following the induction of general anesthesia and paracetamol (1 g IV) at the end of surgery. During the postoperative period, paracetamol (1 g IV) was administered three times a day at 8-h intervals; tenoxicam (20 mg IV) was administered twice a day at 12-h intervals. The PCA device (BodyGuard 575 Pain Manager, UK) was set so that the bolus requested dose was 20 µg/kg, the lock-out time was 6–10 min, and the 4-h limit was 80% of the maximum accessible dose. Where resting VAS was > 3 despite the PCA request, tramadol (100 mg IV, max. 300 mg/day) was administered as a rescue analgesic.

During the postoperative period, recovery was evaluated in three different states: 1, 3, 6, 12, and 24 h at rest (VASr), during coughing (VASc), and at the moment of deep inspiration (VASi). In addition, in the postoperative 3rd month, the patients were contacted by phone, and their resting/coughing/deep inspiration VAS scores were recorded.

### Outcomes

The primary measure was the total morphine consumption within the postoperative 24 h. The secondary measurements were VAS scores at rest, during coughing, and at the moment of deep inspiration during the postoperative 24 h and 3 months, the number of patients needing rescue analgesia, frequency of antiemetic use, intraoperative remifentanil consumption, postoperative first oral intake, first urination, first defecation and first mobilization time, duration of hospitalization, and incidence of pruritus.

### Sample size calculation

Preliminary data from our pilot study of 10 patients per group revealed that the mean postoperative 24-h total morphine consumption was 11.0 ± 1.9 mg for the control group and 9.8 ± 1.5 mg for the ESPB group. According to the power analysis with independent samples *t*-test using IBM SPSS V23, the minimum number of patients to be included in the study according to the confidence interval of 95% (1-α), test power of 80% (1-β), effect size of *d* = 0.701, and the one-way hypothesis was determined as 26 in a group. Taking into account the possibility of data loss or patient dropout, 35 patients were included in each group.

### Statistical analysis

The data were analyzed with IBM SPSS V23. Compliance with normal distribution was examined with the Kolmogorov–Smirnov test. The chi-squared test was used to compare the categorical data by groups. The Mann–Whitney *U*-test was used to compare the data that was not normally distributed according to binary groups. Cochran’s Q test was used to compare the categorical data by intragroup time. Friedman test was used to compare the data that were not normally distributed according to the intragroup time. The results of the analysis were presented as mean ± standard deviation and median (Q1–Q3) for quantitative data and frequency (percentage) for categorical variables. For subgroup analysis, the conformity of the data to the normal distribution was assessed using the Shapiro–Wilk test. The independent two-sample *t*-test was used to compare the normally distributed data of the groups, while the Mann–Whitney *U*-test was used to compare the non-normally distributed data of the groups. The Friedman test was used for data that were not normally distributed to examine the change in the scale scores over time. The chi-squared test and Fisher’s exact test were used to compare the categorical variables of the groups. The results were presented as mean ± standard deviation (95% CI) and median (minimum–maximum) for quantitative data and as frequency (percentage) for categorical variables. The statistical significance level was *p* < 0.05.

## Results

**Fig. 1:**
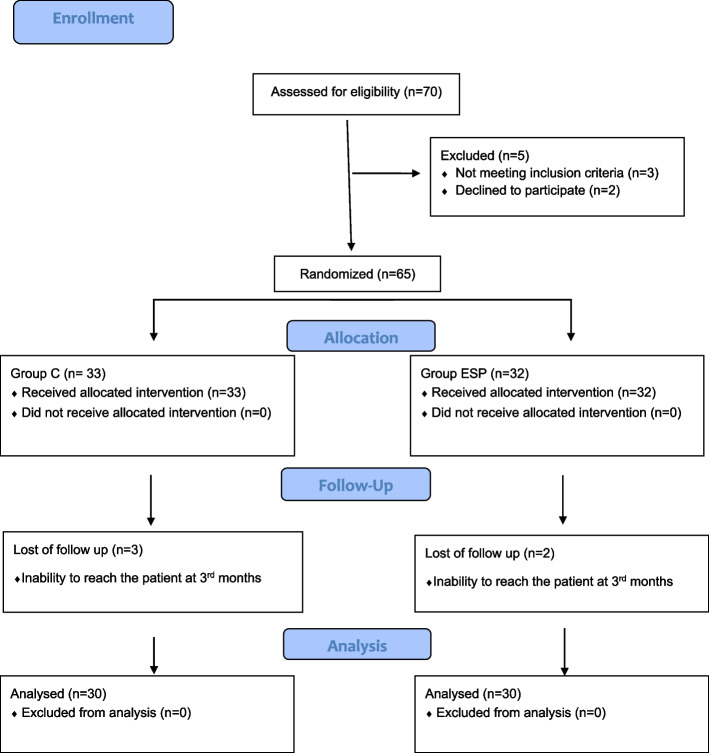
Flow diagram showing the distribution of patient data. Abbreviations: ESP, erector spinae plane

Seventy patients were evaluated for eligibility for the study. Ten participants were excluded from the study, and the results of 60 patients were analyzed, with thirty patients in each group (Fig. 1).

There was no difference between the groups in terms of age, sex, ASA scores, surgical method (laparoscopy vs. laparotomy), and durations (Table 1).

**Table 1:** Patient demographic and surgical characteristics and clinical outcomes

	**Group C (*n* = 30)**	**Group ESP (*n* = 30)**	***p*-Value**
**Age (year)**	58.5 ± 8.1	56.3 ± 8.7	
**Sex**			
Male	19 (63.3)	20 (66.7)	
Female	11 (36.7)	10 (33.3)	
**ASA classification**			
I	14 (46.7)	13 (43.3)	
II	16 (53.3)	17 (56.7)	
**Type of surgery**			
Laparoscopic	13 (43.3)	14 (46.7)	
Open	17 (56.7)	16 (53.3)	
**Operation time (min)**	223.33 ± 44.2	230.07 ± 50.5	
**Intraoperative remifentanil consumption (μg)**	1384 ± 271.39	995.17 ± 308.05	** < 0.001**
**Patients given rescue analgesic in first 24 h**	18 (66.7)	14 (46.6)	> 0.113
**Patients used antiemetic drug**	14 (46.7)	6 (20)	**0.046**
The number of patients who had itching (*PVAS* ≥ 4) at 24 h postoperatively	17 (56.7)	7 (23.3)	**0.008**
**Time to first oral feeding (h)**	58.10 ± 20.75	57.57 ± 11.85	0.206
**Time to first urination (h)**	22.47 ± 11.20	19.00 ± 4.59	0.129
**Time to first flatus (h)**	59.80 ± 12.48	55.23 ± 9.41	**0.037**
**Time to first mobilization (h)**	48.43 ± 22.61	53.70 ± 15.75	0.882
**Length of hospital stay (day)**	8.50 ± 4.25	6.37 ± 2.04	**0.035**

Continuous variables are presented as median (interquartile range, IQR) or mean ± standard deviation, and categorical variables are presented as counts (percentages). Statistically significant difference is highlighted in bold

Abbreviations: *ASA* American Society of Anesthesiologists, *ESP* Erector spinae plane

The cumulative PCA morphine consumption at 2–4 h post-surgery was statistically significantly lower in the ESPB group than in the control group (7.10 mg vs 13.23 mg). It was observed that morphine consumption was higher in the control group than in the ESPB group during the postoperative 1st, 3rd, and 6th h (*p* < 0.05) (Table 2). The sub-group analysis of the amount of morphine consumed in 24 h, which was conducted by considering the surgical method, showed that consumption was higher in the control group (laparoscopy, 11 mg vs. 6.71 mg; laparotomy, 14.94 mg vs. 7.44 mg) (Table 3).

**Table 2:** Comparison of the postoperative morphine consumption between the groups

	**Group C (*n* = 30)**		**Group ESP (*n* = 30)**		***p*-Value**
	Mean ± SD (% 95 *CI*)	Median [Q1-Q3]	Mean ± SD (% 95 *CI*)	Median [Q1-Q3]	
**1st h**	5.40 ± 2.24 (4.56–6.24)	6.00 [4-8]	3.93 ± 1.86 (3.24–4.63)	4.00 [2-4]	**0.013**
**3rd h**	3.93 ± 2.13 (3.14–4.73)	4.00 [4-6]	2.13 ± 1.48 (1.58–2.69)	2.00 [2-2]	**0.001**
**6th h**	2.73 ± 2.00 (1.99–3.48)	2.00 [2-4]	0.67 ± 1.32 (0.17–1.16)	0.00 [0-2]	** < 0.001**
**12th h**	0.93 ± 1.89 (0.21–1.65)	0.00 [0-2]	0.33 ± 1.18 (0.11–0.78)	0.00 [0-0]	0.053
**24th h**	0.28 ± 0.65 (0.03–0.52)	0.00 [0-0]	0.03 ± 0.18 (0.04–0.10)	0.00 [0-0]	0.073
**Total**	13.23 ± 3.98 (11.75–0)	12.5 [8-14]	7.10 ± 2.6 (6.13–0)	8 [6-10]	** < 0.001**

Note: Data are expressed as mean ± standard deviation (95% CI) and median ([IQR, Q1–Q3]). Statistically significant difference is highlighted in bold

Abbreviations: *IQR* Interquantilerange, *CI* Confidence interval, *ESP* Erector spinae plane

**Table 3:** Comparison of the postoperative morphine consumption in the first 24 h according to the surgical technique

	**Group C (*n* = 30)**		**Group ESP (*n* = 30)**		***p*-Value**
	Mean ± SD (% 95 *CI*)	Median [Q1-Q3]	Mean ± SD (% 95 *CI*)	Median [Q1-Q3]	
**Laparoscopic**	11.00 ± 2.94 (9.22–12.78)	10 [10-12]	6.71 ± 3.00 (4.98–8.45)	6 [4-10]	**0.001**
**Open**	14.94 ± 3.88 (12.95–16.94)	14 [12-16]	7.44 ± 2.25 (6.24–8.64)	8 [6-8]	** < 0.001**
**Total**	13.23 ± 3.98 (11.75–0)	12.5 [8-14]	7.10 ± 2.6 (6.13–0)	8 [6-10]	** < 0.001**

Note: Data are expressed as mean ± standard deviation (% 95 CI) and median ([IQR, Q1–Q3]). Statistically significant difference is highlighted in bold

Abbreviations: *IQR* Interquantilerange, *CI* Confidence interval, *ESP* Erector spinae plane block

Although the number of patients who needed rescue analgesia was higher in the control group than in the ESPB group, the difference was not statistically significant. The intraoperative total remifentanil consumption was statistically significantly higher in the control group than in the ESPB group. The antiemetic requirement of the patients in the control group was higher than that of the patients in the ESPB group. The time to first defecation and duration of hospitalization were significantly longer in the control group than in the ESPB group. While the times to first oral intake, first urination, and first mobilization were similar in the two groups, the percentage of patients with itching was higher in the control group than in the ESPB group (Table 1).

**Fig. 2:**
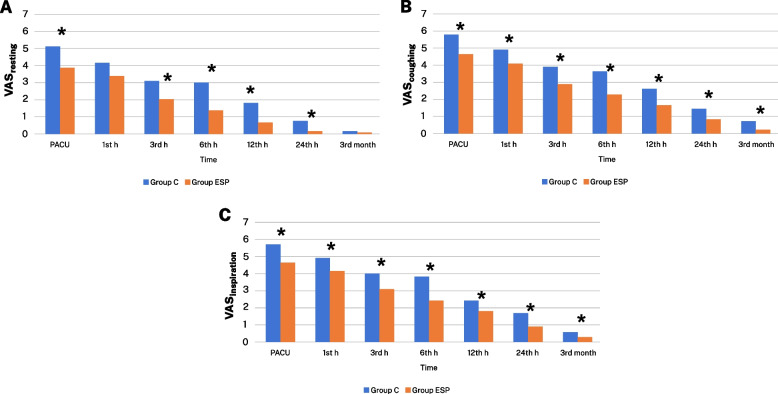
**A** VAS_resting_ scores of the groups at different time points. **B** VAS_coughing_ scores of the groups at different time points. **C** VAS_inspiration_ scores of the groups at different time points. Data are expressed as mean ± standard deviation. Abbreviations: VAS, visual analog scale; PACU, postanesthesia care unit; ESP, erector spinae plane. *p < 0.05 statistically significant according to group ESP

While the resting VAS scores in the postanesthesia care unit (PACU) and the postoperative 3-, 6-, 12-, and 24-h measurements were higher in the control group than in the ESPB group, no difference was observed between the measurements during the 3rd month. The VAS scores at the time of coughing/deep inspiration were higher for the control group at all measurement times (Fig. 2).

Considering the surgical method, there was no difference between the VAS scores for the laparoscopic cases in the groups (except for the resting VAS at the 6th h). For the laparotomy cases, only the cough VAS score was higher in the control group in the 3rd month, although the first 24-h VAS scores (except for the 6- and 12-h deep inspiration VAS) were higher in the control group. There were no opioid-related adverse events (itching, bladder globe, etc.) and block-related (hematoma formation, pneumo/hemothorax, infection or local anesthetic systemic toxicity, etc.) complications.

## Discussion

In the present study, except for the 3rd month resting VAS scores, ESP block was effective for pain scores and decreased early postoperative morphine consumption in colorectal surgeries. The intraoperative remifentanil consumption, pruritus, and postoperative antiemetic requirement were also lower. The time to first defecation and duration of hospitalization were shorter in patients with the block.

According to reports of meta-analyses in the literature, ESPB effectively reduces postoperative opioid consumption [7–10]. When Kendall et al. reviewed 12 studies on ESPB, they found that ESPB reduced postoperative opioid consumption by approximately 8.84 mg [7], while this amount was 7.13 mg in the present study. However, a closer look at this meta-analysis showed that the studies were quite heterogeneous, and the decrease in opioid consumption in the sub-group analysis was notable after the orthopedic surgical procedures of the chest and spine. In contrast, no significant difference was found for abdominal surgeries. Considering the other studies in the literature, this may be associated with the block level and the difference in drug doses. While a decrease in opioid consumption is observed at all measurement times during 24 h with blocks applied at the T2 [11], T4 [12], and T5 [13] levels with 20 ml of 0.25% bupivacaine, it may not be achieved at lower levels (T9–L3) [14, 15]. Similarly, in the present study, ESPB applied with 20 ml of 0.25% bupivacaine at the T9 level decreased opioid consumption only within the first 6 h. Likewise, Tulgar et al. observed that opioid consumption decreased only within the first 12 h postoperatively after the block (laparoscopic cholecystectomy) they performed using 20 ml of 0.375% bupivacaine at the T9 level [15]. On the other hand, after the block applied by Çiftçi et al. with 20 ml of 0.25% bupivacaine at the L3 level, a decrease in postoperative 24-h opioid consumption was observed [16]. A general review of the literature on ESPB reveals a substantial inconsistency in the results. The opioid-sparing effect of ESPB applied at the higher levels may have lasted longer than the block applied at the lower levels because the surgeries performed were different (for example, mastectomy, lumbar, and colorectal surgery).

The reasons for such inconsistency in the ESPB results in the literature, other than the block level, may include the following: differences in the drug volumes/concentrations and surgical types and methods (L/S vs. open surgery) applied during ESPB in the studies, which would result in differences in pain intensity and duration, and the differences in the multimodal analgesia protocol used [17–19]. As a consequence, the high heterogeneity of the studies prevents us from reaching definite conclusions about ESPB.

It was observed that postoperative pain scores were lower in the block group in the present study, which is consistent with reports in the literature [7, 8, 10, 20]. It has been reported that pain following colorectal surgeries may persist after the acute phase [21]. In their series of 624 patients, Jin et al. [21] found that pain scores were higher at 3 months in one of every three patients and at 6 months in one out of every five patients who underwent colorectal surgery. In the present study, we found that the VAS scores in the 3rd month were lower in the block group. The reason for the persistence of the pain for long after these surgeries has not yet been fully elucidated; however, sensitization plays an important role [22]. The suppression of inflammation during the early period, prevention of peripheral and central sensitization, and using minimally invasive surgical methods may help prevent persistent pain after the acute phase. Effective postoperative pain relief is one of the important steps in ERAS protocols, and it is recommended that facial plane blocks should be added to multimodal analgesia regimens [2].

In the present study, the total intraoperative remifentanil dose consumed was lower in the block group. This suggests that performing the block before surgery decreases intraoperative opioid consumption [23]. Although there are studies in the literature demonstrating that the need for postoperative rescue analgesia decreases with ESPB [15, 24, 25], the number of patients needing rescue analgesia in the present study was comparable between the groups.

There are few studies in the literature related to ESPB presenting data on postoperative pruritus. In the study of Fu et al. [20], the incidence of pruritus in the patients who underwent ESPB was lower, although not statistically significant. In the present study, postoperative pruritus and antiemetic requirement were significantly lower in the ESPB group. This result may be due to the decreased need for opioids in the group which administered the block.

Postoperative ileus occurs in 25% of patients following colorectal surgery [26], and one of the most important reasons for prolonged hospital stay is delayed optimization of bowel function [27]. A decrease in patient discomfort and shortening of hospital stay as a result of the prevention of ileus are one of the important components of ERAS protocols. A decrease in opioid use is associated with improved recovery quality and shorter time to mobilization and hospital stay [24, 28]. In the present study, the time to first defecation and duration of hospitalization were shorter in the ESPB group. Similarly, ESPB shortened hospital stay after laparoscopic surgeries in the study of Özdemir et al. [29].

### Limitations

The first limitation of our study can be considered as the application of two different surgical methods (laparoscopy/laparotomy) to the groups. The second limitation may be the fact that the sham block group was not created due to ethical concerns. In addition, our long-term pain assessments were carried out by contacting the patients by phone, and we did not use a validated assessment scale (such as *douleur neuropathique en* 4 questions or the brief pain inventory). The last limitation is that patients were not blinded to the study due to the study design.

## Conclusion

In conclusion, ESPB decreased postoperative pain scores and the need for opioids in colorectal surgeries, as well as decreased itching, incidence of antiemetic use, and length of hospital stay.

## Data Availability

The datasets used and/or analyzed during the current study are available from the corresponding author on reasonable request.

## Abbreviations

ASA: American Society of Anesthesiologists
ERAS: Enhanced recovery after surgery
ESPB: Erector spinae plane block
PACU: Postanesthesia care unit
PCA: Patient-controlled analgesia
PONV: Postoperative nausea and vomiting
VAS: Visual analog scale

## References

[1] Ates I, Disci E, Yayik AM (2022). (2022) Ultrasound-guided erector spinae plane block versus trocar site local anesthetic infiltration for laparoscopic colorectal resection: a prospective, randomized study. Ann Med Res.

[2] Gustafsson UO, Scott MJ, Hubner M, Nygren J, Demartines N, Francis N, Ljungqvist O (2019). Guidelines for perioperative care in elective colorectal surgery: enhanced recovery after surgery (ERAS®) society recommendations: 2018. World J Surg.

[3] Hannig KE, Jessen C, Soni UK, Børglum J, Bendtsen TF (2018). Erector spinae plane block for elective laparoscopic cholecystectomy in the ambulatory surgical setting Case reports in anesthesiology.

[4] Wildsmith JA (2012). (2022) Continuous thoracic epidural block for surgery: gold standard or debased currency?. Br J Anaesth.

[5] Forero M, Adhikary SD, Lopez H, Tsui C, Chin KJ (2016). The erector spinae plane block: a novel analgesic technique in thoracic neuropathic pain. Reg Anesth Pain Med.

[6] Jones JH, Aldwinckle R (2020). (2020) Interfascial plane blocks and laparoscopic abdominal surgery: a narrative review. Local Reg Anesth.

[7] Kendall MC, Alves L, Traill LL, De Oliveira GS (2020). (2020) The effect of ultrasound-guided erector spinae plane block on postsurgical pain: a meta-analysis of randomized controlled trials. BMC Anesthesiol.

[8] Koo C-H, Hwang J-Y, Shin H-J, Ryu J-H (2020). (2020) The effects of erector spinae plane block in terms of postoperative analgesia in patients undergoing laparoscopic cholecystectomy: a meta-analysis of randomized controlled trials. J Clin Med.

[9] Cui Y, Wang Y, Yang J, Ran L, Zhang Q, Huang Q, Yang X (2022). The effect of single-shot erector spinae plane block (ESPB) on opioid consumption for various surgeries: a meta-analysis of randomized controlled trials. J Pain Res.

[10] Daghmouri MA, Akremi S, Chaouch MA, Mesbahi M, Amouri N, Jaoua H, Ben Fadhel K (2021). Bilateral erector spinae plane block for postoperative analgesia in laparoscopic cholecystectomy: a systematic review and meta-analysis of randomized controlled trials. Pain Pract.

[11] Aksu C, Kuş A, Yörükoğlu HU, TOR KILIÇ C, GÜRKAN Y (2019). Analgesic effect of the bi-level injection erector spinae plane block after breast surgery: A randomized controlled trial. Agri.

[12] Gürkan Y, Aksu C, Kuş A, Yörükoğlu UH, Kılıç CT (2018). (2018) Ultrasound guided erector spinae plane block reduces postoperative opioid consumption following breast surgery: a randomized controlled study. J Clin Anesth.

[13] Ciftci B, Ekinci M, Celik EC, Tukac IC, Bayrak Y, Atalay YO (2020). Efficacy of an ultrasound-guided erector spinae plane block for postoperative analgesia management after video-assisted thoracic surgery: a prospective randomized study. J Cardiothorac Vasc Anesth.

[14] Yayik AM, Cesur S, Ozturk F, Ahiskalioglu A, Ay AN, Celik EC, Karaavci NC (2019). Postoperative analgesic efficacy of the ultrasound-guided erector spinae plane block in patients undergoing lumbar spinal decompression surgery: a randomized controlled study. World neurosurgery.

[15] Tulgar S, Kapakli MS, Senturk O, Selvi O, Serifsoy TE, Ozer Z (2018). (2018) Evaluation of ultrasound-guided erector spinae plane block for postoperative analgesia in laparoscopic cholecystectomy: a prospective, randomized, controlled clinical trial. J Clin Anesth.

[16] Ciftci B, Ekinci M, Celik EC, Yayik AM, Aydin ME, Ahiskalioglu A (2020). Ultrasound-guided erector spinae plane block versus modified-thoracolumbar interfascial plane block for lumbar discectomy surgery: a randomized, controlled study. World Neurosurgery.

[17] Sommer M, De Rijke JM, Van Kleef M, Kessels AGH, Peters ML, Geurts JWJM, Marcus MAE (2008). The prevalence of postoperative pain in a sample of 1490 surgical inpatients. Eur J Anaesthesiol.

[18] Kot P, Rodriguez P, Granell M, Cano B, Rovira L, Morales J, De Andrés J (2019). The erector spinae plane block: a narrative review. Korean J Anesthesiol.

[19] De Cassai A, Bonvicini D, Correale C, Sandei L, Tulgar S, Tonetti T (2019). Erector spinae plane block: a systematic qualitative review. Minerva Anestesiol.

[20] Fu J, Zhang G, Qiu Y (2020). (2020) Erector spinae plane block for postoperative pain and recovery in hepatectomy: a randomized controlled trial. Medicine.

[21] Jin J, Chen Q, Min S, Du X, Zhang D, Qin P (2021). Prevalence and predictors of chronic postsurgical pain after colorectal surgery: a prospective study. Colorectal Dis.

[22] Ji RR, Nackley A, Huh Y, Terrando N, Maixner W (2018). Neuroinflammation and central sensitization in chronic and widespread pain. Anesthesiology.

[23] Fletcher D, Martinez V (2014). (2014) Opioid-induced hyperalgesia in patients after surgery: a systematic review and a meta-analysis. Br J Anaesth.

[24] Karaca Ö, Pınar HU (2020). (2020) Efficacy of ultrasound-guided erector spinae ID plane block for postoperative analgesia in laparoscopic cholecystectomy: a retrospective cohort study. Anestezi Dergisi.

[25] Elyazed MMA, Mostafa SF, Abdelghany MS, Eid GM (2019). (2019) Ultrasound-guided erector spinae plane block in patients undergoing open epigastric hernia repair: a prospective randomized controlled study. Anesth Analg.

[26] Keller D, Stein SL (2013). (2013) Facilitating return of bowel function after colorectal surgery: alvimopan and gum chewing. Clin Colon Rectal Surg.

[27] Moningi S, Patki A, Padhy N, Ramachandran G (2019). (2019) Enhanced recovery after surgery: an anesthesiologist's perspective. J Anaesthesiol Clin Pharmacol.

[28] Finnerty DT, Buggy DJ (2021). (2021) Efficacy of the erector spinae plane (ESP) block for quality of recovery in posterior thoraco-lumbar spinal decompression surgery: study protocol for a randomised controlled trial. Trials.

[29] Ozdemir H, Araz C, Karaca O, Turk E (2022). Comparison of ultrasound-guided erector spinae plane block and subcostal transversus abdominis plane block for postoperative analgesia after laparoscopic cholecystectomy: a randomized, controlled trial. J Invest Surg.

